# Ethnobotanical study of underutilized wild edible plants and threats to their long-term existence in Midakegn District, West Shewa Zone, Central Ethiopia

**DOI:** 10.1186/s13002-023-00601-8

**Published:** 2023-07-14

**Authors:** Sheleme Guzo, Ermias Lulekal, Sileshi Nemomissa

**Affiliations:** 1 Department of Plant Biology and Biodiversity Management, College of Natural and Computational Science, Addis Ababa University, 1176 Addis Ababa, Ethiopia; 2 Department of Botanical Science, College of Natural Science, Jimma University, 378, Jimma, Ethiopia

**Keywords:** Ethnobotany, Ethiopia, Midakegn, Underutilized wild edible plants

## Abstract

**Background:**

Ethiopia is endowed with much plant diversity. The insignificant number of studies on wild edible plants with their ethnobotanical perspectives indicated that this plant diversity comprised only hundreds of wild edible plants used to supplement food sources for the local community under different conditions. There still need to be further investigations throughout the country when compared to the total area and cultural diversity of the country. However, they are seriously under pressure due to different natural and human influences. Therefore, the study was conducted to document underutilized wild edible plants along with their associated indigenous knowledge and explore threats to them in Midakegn District.

**Methods:**

A questionnaire survey, semi-structured interviews, a market survey, score ranking, and focused group discussions were employed for data collection. Statistical analysis of ethnobotanical knowledge mean variation between different informant groups was computed by using one-way ANOVA in the IBM SPSS Statics version 24 package.

**Results:**

A total of fifty underutilized wild edible plants belonging to 39 genera and 30 families were collected, recorded, and documented. The families *Moraceae* (four), *Fabaceae*, *Flacourtaceae*, *Myrtaceae*, *Rosaceae*, and *Tiliaceae* (each three) represented the highest number of species. It comprised shrubs (44%), trees (36%), herbs (18%), and epiphytes (2%). Fruits (62.3%) were found to be the most frequently used and mostly taken raw, fresh, or dried. These edible resources were consumed to supplement staple foods (67.3%), whereas 25% were used as emergency foods. The majority of species (96%) had multiple uses in addition to their edibility. A significantly higher (*P* < 0.05) number of underutilized wild edible plants were cited by males than females, by key informants than generals, elders than youngsters, illiterate than literate, and poorer than other wealth class groups of the community. Priority rankings indicate that agricultural expansion, fuel wood harvest, overgrazing, and selective harvesting are the most threatening factors to underutilized wild edible plants.

**Conclusions:**

Fifty underutilized wild edible plants, along with their associated indigenous knowledge, were recorded. Local people utilize them for supplementing staple food, as emergency food, to get relief, trust, and chew during drought. But they are mainly threatened by different human activities in the study area.

## Introduction

In human history, plant wealth of about 40,000–100,000 species has been used by humans for multiple purposes [1, 2], and this globally accounts for about 5% of the total plant species of the world [3]. Of these, 30,000 species so far have been identified as edible [4], and about 7000 species have been cultivated and/or collected for food at one time or another [1]. As a result of the Green Revolution, many of these local, traditional crop species and varieties have been replaced by high-yielding staple crop cultivars developed by modern breeding programs [5].

The flora of Ethiopia has been endowed with an estimated 7000 higher plants [6]. Of these, nearly 3% (203 species) were reported as wild edible plants used to supplement food sources in different parts of the country under different conditions [3]. After ten years of this report, the review report done on wild edible plants in Ethiopia indicated the country has 413 WEPs with their ethnobotanical information, and this accounts for about 5% of the higher plant species [7]. Rural communities in the country depend on these edible resources to meet their food needs, mostly during periods of food crises [8]. During the drought-stricken years of 1966–1969, the Konso people of southern Ethiopia survived by increasing their consumption of wild food plants [9]. In another country, the consumption of foods from the wild also played a significant role in saving lives during times of hunger. For instance, in 1973 and 1984–1985, the Berti people of Sudan relied on tree fruits and wild grass seeds to survive severe food shortages [10]. Consumption of such edibles contributes essential nutrients that play a vital role in maintaining food security. Consumption of wild green leafy vegetables as part of supplements or main dishes by the southern population in Ethiopia helps to alleviate malnutrition [11]. Thus, for community members who are vulnerable to malnutrition, particularly children, consumption of wild edible plants helps them get the essential fats, proteins, vitamins, and minerals they need [10].

Ensuring household food security is not the only importance of those plant diversities; they also contain and provide materials for economic, medicinal, and forage values and also possess and preserve cultural heritages, biological information, and indigenous knowledge about their utility [6]. With such a variety of plants, people offered and used materials for income generation, medical care, foraging, fuel wood, construction, honey production, and detergent [12]. In most cases, rural people have deep, non-similar indigenous knowledge of wild edible plants, and their consumption is still an integral part of the different cultures in the different parts of the country under different conditions [6, 9]. However, many of these plants are underutilized and/or neglected by indigenous farming communities for specific socioeconomic reasons [13]. For example, 33 underutilized wild edible plants in the local community were reported in the Chilga District, northwestern Ethiopia [12].

Even though there is a lack of consensus on the definition, a wide range of terms are used for underutilized plant species, which include minor, neglected, local, traditional, underexploited, underdeveloped, orphan, lost, new, promising, and alternative plant species [4, 14, 15]. Of these, the most widely used term is underutilized, which refers to plant species that communities traditionally use for food, fiber, animal fodder, oil, or medicine, but that have further undeveloped potential uses [4]. They are less represented in ex situ germplasm collections [1], their potential has not been fully realized [16], and they are not included in the official farming system [17]. Those plant species are currently maintained through in situ conservation [13], and this is because they are less competitive with other species in the same agricultural environment [18]. However, UWEPs are still important resources for the subsistence of local communities, socio-cultural preferences, and traditional uses. They could be serving as an alternative food source, while the world faced critical challenges due to climatic change, food security, human nutrition, and overdependence on a few staple crops for the world food supply [13, 18]. These species could become important crops to reduce risks and, adapt to shocks caused by climate change in the future [13, 19]. They significantly improve health and nutrition, livelihoods, household food security, and the ecological sustainability of the environment [4, 6, 16]. Especially in the case of nutritional value, wild and semi-wild foods provide a diversity of nutrients in the diets of many households [17].

There are four major areas where underutilized species can make significant contributions to sustainable agriculture: food security and better nutrition; increased income for the rural poor; ecosystem stability; and cultural diversity associated with local food habits and religious and social rituals [16, 18]. Their role has evolved over time, and as it is today, it adds to the quality of life besides meeting the needs of the rural poor in particular [4], and it becomes extremely important to mitigate risks and adapt to shocks caused by climate changes. Thus, the genetic resources of UWEPs might become more attractive to farmers [13]. This might be due to their greater potential to cope up with the adverse effects encountered due to extreme climate change than conventional crops [20]. Specifically, in developing countries, there is a wider opportunity to use these species to ensure food security, and they could be important crops in the future [13, 21].

Thus, giving attention to UWEP species is an effective way to maintain diverse and healthy diets, and mainstreaming them into local food systems could help to combat malnutrition, particularly among low-income rural households and the more vulnerable social groups in developing countries [1, 21]. Besides multiple use categories, it provides synthesized information on underutilized grains, roots and tubers, leafy vegetables, fruits, spices, condiments, etc. It also brings out several emerging concerns for their further promotion of human welfare in addressing food security, minimizing malnutrition, poverty reduction, and income generation. What is equally important is the need to capture the associated indigenous knowledge base held by the indigenous community. For a wide range of such under-cultivated species, there is an urgent need to broaden this base of species effectively and sustainably to protect and enhance the use of such regionally significant species that are also more globally applicable in agricultural and environmental management. Missing attention might lead to the erosion of the available gene pools in their areas of diversity and cultivation.

Ethiopian farmers face common challenges due to deforestation, drought, land degradation, and climate change, and they experience significant food insecurity [22, 23]. The situation is also very common in the study area, the central part of Shewa, and the households are similarly facing challenges. Under such circumstances, underutilized and/or neglected plants can offer alternatives to the basic crops to ameliorate the situation and maintain farm productivity. This is due to their natural adaptability and resistance to challenges growing due to various environmental constraints [24]. Future local and global food security could greatly benefit from such species [13, 19, 24]. On the other side, this central part, which includes the study area, is one of the most densely populated areas of the country. The wild edible plants and the associated indigenous knowledge with their usage are in danger due to human and natural impacts. Thus, the study area at the local level and Ethiopia at the country level would benefit from such research to increase the production and value of these resources and promote their widespread cultivation, which would increase food, economic, and nutritional security.

On the other hand, only an insignificant number of investigations on wild edible plants with their ethnobotanical perspectives were reported throughout the country when compared to its total area and cultural diversity [7, 9]. This suggests that the ethnobotanical knowledge of UWEPs was inadequately documented and is still held in the collective memory of senior community members. The term "UWEPs" used in this study revealed that all wild edible plants and their potential values got less attention and thus remained underestimated among the indigenous people of the study area. Giving poor attention to such plants meant that indigenous communities missed the opportunity to access rich nutrients and health-promoting compounds with preventive effects against malnutrition and some chronic diseases [21]. The literature survey carried out on the ethnobotanical studies showed that there has been no previous investigation reported on UWEPs and the associated indigenous knowledge of the local people in the Midakegn District. Therefore, the purposes of this study were to: (1) collect UWEPs and document the associated ethnobotanical knowledge of the local people residing in the study area before it is lost forever and (2) explore the threat of underutilized wild edible plants in the study area. This helps to promote indigenous knowledge on the potential utilization of UWEPs and their importance for present and future socio-economic significance.

## Material and methods

### Description of the study area

**Fig. 1:**
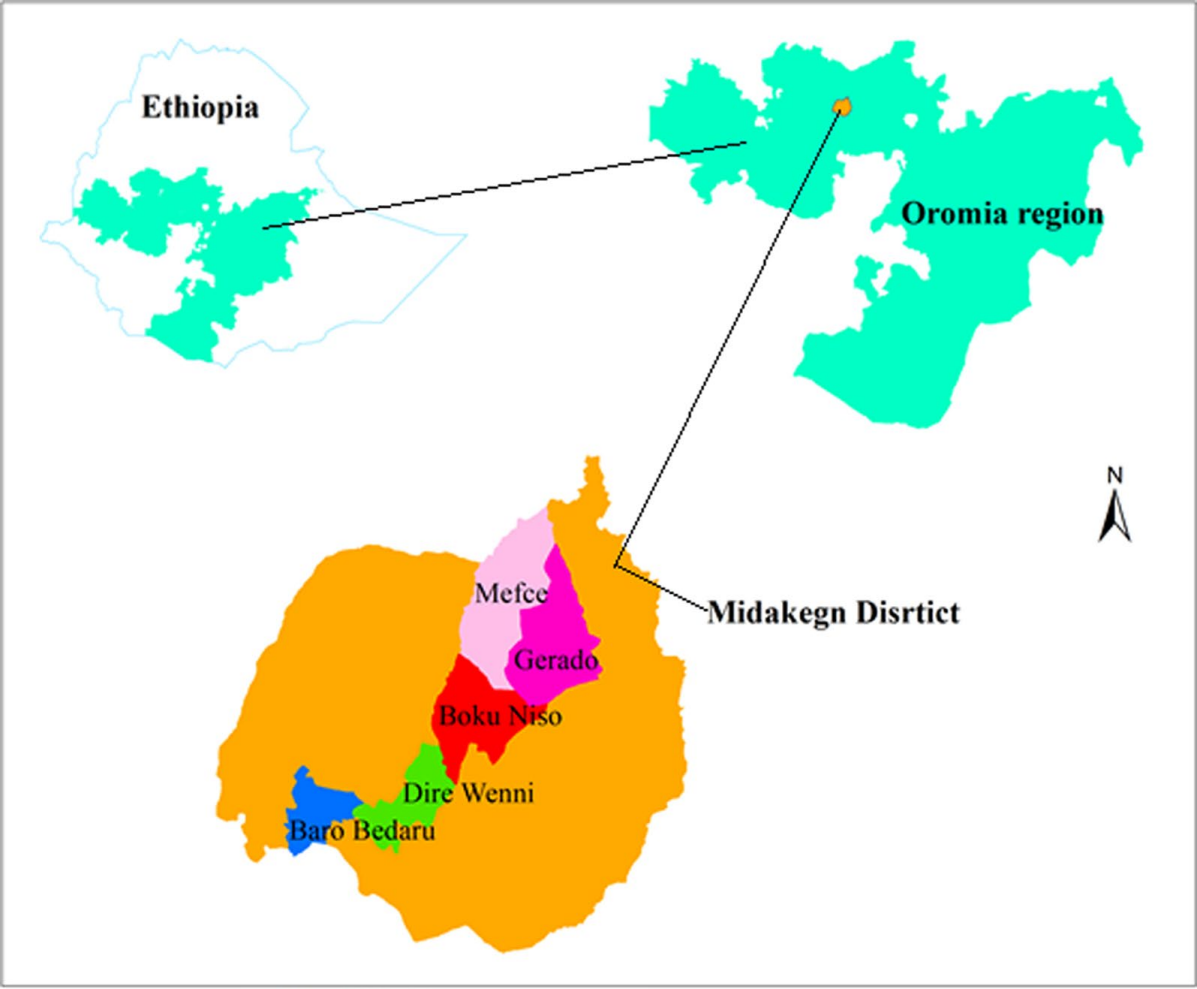
A map of Ethiopia shows the Oromia region and the study district with study sites

Midakegn District is one of the administrative districts forming the West Shewa Zone of Oromia Regional State, in Central Ethiopia (Fig. 1). The study area lies between 09° 02′ 57″ and 09° 23′ 45″ N latitudes and 037° 23′–037° 45′ 54″ E longitudes, with a total area of about 91,051 hectares (ha) and an elevation range of 1290–3058 m above sea level. According to the Midakegn District Agricultural and Rural Development Office's (MARDO) 2020 annual report (unpublished), the district is known for having three agro-climatic zones. These are namely: “*Baddaa*:” (highland) covers 12.5% of the western part of the “*Balemi*” (the capital town of the district); “*Badda Daree*” (middleland) covers 50% by extending eastward to the northwest through the central part of the district; and “*Gammojji*” (lowland) covers the remaining 37.5% in the northern part. The study area faces a humid air current coming from the Atlantic Ocean and receives heavy rainfall during the main rainy season (May to September). The highest mean annual rainfall of the study area within ten years was 186.4 mm, recorded in July, followed by 183.2 mm in August, whereas the lowest mean total was 6.5 mm, recorded in December. The lowest mean temperature over ten years was 8.7 °C recorded in December, whereas the highest was 24.6 °C recorded in February. The total population of the district was about 106,438 people, of whom 52,148 (49%) were males and 54,290 (51%) were females. Subsistence mixed agriculture was the economic mainstay for the population of the study area.

### Site selection

After being informed of the purpose of the field study, the district authorities requested permission and granted it. In order to gather broad information for site selection, a reconnaissance survey and contacts with specialists, including district and kebeles' officials, agricultural workers, and elders, were made between March 1 and 15, 2021. Then, five kebeles (the lowest administrative unit in Ethiopia) from the three agro-ecological zones were chosen for UWEPs data collection: one from the highland, two from the midland, and two from the lowland (Table 1). These kebeles were chosen based on criteria including the degree of accessibility for data collection, such as the varied altitudinal location, availability of UWEP, and livelihood of the local people.

**Table 1:** Sampled kebeles, number of respondents, and agroecologies of the study area

Study kebeles	Key informants	General informants	Agroecology
Baro Bidaru	5	68	Highland
Dire wenni	5	66	
Boku Niso	5	66	Midland
Mafce	5	66	
Gerado	5	67	Lowland
Total	25	333	

### Key informant and household selection

The snowball and a simple stratification method were used in this study to choose the informants. This was used to analyze the indigenous knowledge and plant utilization differences among the local communities in the study area. Following [25], key informants (KIs) were chosen using the snowball method. The approach was used to identify the research area's most knowledgeable individuals. According to this study, KIs are those who are relatively most aware of UWEPs and the regional circumstances in the study area than other residents. Therefore, a total of 25 KIs (five from each kebele) were chosen. In the beginning, individually, ten knowledgeable people in each kebele were called up by three elders who were chosen at random. Then, the five most knowledgeable individuals who were often informed by the three farmers were selected as KIs and took part in preliminary ethnobotanical data collection, a survey questionnaire, a semi-structured interview, a scoring and ranking procedure, and focus group discussions. A simple stratification technique was applied to select general informants (GIs) from the households residing at the study sites. The purpose of stratification was also to involve all age and wealth categories during data collection. Therefore, by following [26], households were categorized by age (18–40 years and > 40 years) and wealth class (poor, medium, and rich). The Yamane formula, as described in [27], was used to determine the sample size from the total number of households at the study site at a 95% confidence level, *P* = 0.5, and ± 5% level of precision. Thus, a total of 358 informants (25 KIs and 333 GIs) out of 3478 households belonging to the study sites were selected, and one person from each representative household was involved in survey question activities carried out during data collection.

$$n= \frac{N}{1+N{(e}^{2})}$$

where *n* is the sample size representative, *N* is the total households, *e* is the level of area precision, and 1 is the probability of an event occurring.

### Data collection and analysis

To collect ethnobotanical data, questionnaires and semi-structured interviews were prepared, pretested, and administered to households (general) and KIs as stated in [26]. Thus, the same standard open- and closed-ended questions were prepared in English and translated into the local language, “*Afan Oromo*,” for the interview. After the objectives of the current study were shared with the interviewers, an interview was administered to each identified respondent by the researcher on a face-to-face and one-on-one basis based on their consent at the place of their choice, which could be at home or in the field. Consequently, field observations were conducted by guided field walks to identify where UWEPs are grown and to collect their specimens. Detailed information targeting UWEPs known to the informants, including the plant species used, vernacular names, part(s) used, method of preparation, ways of consumption, consumption role, other use diversity, threatening factors, and conservation strategies, was collected and recorded. All UWEPs listed in the socio-economic survey were verified, and ideas that deviated from reality were removed from the data. Finally, all encountered plants were collected, pressed, dried, and recorded by their vernacular names along with a voucher number. Preliminary specimen identification was attempted in the field and confirmed at the National Herbarium (ETH) by using published volumes of the Flora of Ethiopia and Eritrea [28–35] and comparison with authentic specimens, illustrations, taxonomic keys, and with the assistance of experts at the National Herbarium (ETH), AAU. Market surveys at three local marketplaces (Balemi, Wayilo, and Bitile) were conducted in the study area. The main purpose of the market survey was to record the marketability of the edible parts and kinds of UWEPs sold in the study area. Thus, a semi-structured interview with edible part sellers and informants and participatory observation were conducted to assess the variety and marketability of the plants.

The collected ethnobotanical information was organized and analyzed by Microsoft Excel 2010 spreadsheet software. The difference in ethnobotanical knowledge between informant groups was computed by one-way ANOVA in the IBM SPSS Statics version 24 package to check the existence of significant differences (at a 95% confidence level) between means.

Preference ranking for more popular or palatable UWEPs, direct matrix ranking for multipurpose UWEPs, and priority ranking of threats to UWEPs were done as follows [25, 26]. In the preference ranking process, the values (0–5) were given by KIs, and each value was summed up, and the average was taken to determine the preference of one over the other. A direct matrix ranking was conducted for ten multipurpose UWEPs commonly and frequently reported in the study district. The purpose is to assess their relative importance to the local people and the extent of the existing threats related to their use values. Based on the service categories, 10 KIs were asked to assign use values for each attribute. The list of these attributes included medicinal, fuel wood (firewood and charcoal), construction, farm and household tools, fodder, live shade, live fence, honey bee forage, and soil and water conservation. The use values (0–5) were given. The average use values given for each multipurpose species in each use category by each KI were recorded, and the values were summed up for each species and ranked. Priority ranking on recorded major threatening factors to these plants was also done by 10 KIs based on their degree of destructive effects. Values (1–6) were given, all values given by each KI were summed up to report the most concerning factor.

## Results and discussion

### Taxonomic diversity of underutilized wild edible plants in the study area

In this study, a total of 50 species of UWEPs distributed into 39 genera and 30 families were gathered and documented from the study area (Table 2). All the UWEPs were reported by the local community with their vernacular names. Out of these total reports, 36 species (72%), 25 species (50%), 22 species (44%), 21 species (42%), 18 species (36%), 17 species (34%), and 13 species (26%) were reported in the study conducted by [3] at the country level, by [36] in Soro District, southern Ethiopia, by [37] in Berehet District, North Shewa Zone of Amhara Region, by [38] in Muja District, northwestern Ethiopia, by [11] in southern Ethiopia, by [39] in West Gojjam, Ethiopia, and by [40] in Mieso District, eastern Ethiopia, as wild edible plant species, respectively, and 12 species (24%) were reported in a similar ethnobotanical study conducted by [12] in the Chilga District, northwestern Ethiopia. The number of UWEPs recorded in the study area was lower than the number of wild edible plants reported by [6, 11, 36] in other parts of Ethiopia. But it was greater than the number of wild edible plants reported in several ethnobotanical studies (e.g., [12, 38–42]) and compared to the number of species reported as wild edibles in other regions of Ethiopia [37, 43, 44]. The relatively higher number indicates that the catchment under study was generally endowed with diverse and rich sources of UWEPs with their associated indigenous knowledge, and such high diversity might be due to the existence of different agroecological zones in the study area. The possible variation among different localities of the country could be due to the existence of variations in community culture, vegetation cover, the size of the study area, and environmental conditions. After evaluating the country's endemic plant report [45], and through assessing the ethnobotanical wild edible plants review report at country level by [7] and other similar study reports in the country, out of the total documented species, two species (*Impatiens rothii* Hook. f. and *Urtica simensis* Steudel) were endemic to the country, and three species, *Canarina eminii* Asch. and Schweinf, *Gardenia terniffolia* Schumach and Thonn, and *I. rothii,* which were not previously known as edible plants, were newly discovered and added to the wild edible database of the country.

**Table 2:** Underutilized wild edible plants collected from Midakegn District

Scientific name	Family	Vernacular name	H	Hb	Pu	Mode of consumption	Vn
*Vachellia abyssinica* (Hochst. ex Benth.) Kyal. and Boatwr	Fabaceae	Lafto	T	a, b, c, f	G	Raw, chewing	Mk013
*Faidherbia albida* (Delile)* A. Chev*	Fabaceae	Garbi	T	a, b, c, e, f	G, Se	Raw, chewing Cooked	Mk071
*Acokanthera schimperi* (A.DC.) Schweif	Apocynaceae	Kararu	S	a	F	Raw, rippen	Mk082
*Amaranthus hybridus* L	Amaranthaceae	Lamoyi	H	b, e	L, St	Cooked	Mk001
*Oldeania alpina* (K. Schum.) Stapleton	Poaceae	Lemana	H	g, h	Y	Cooked	Mk032
*Canarina eminii* Asch. and Schweinf	Campanulaceae	Tuxo	E	i	Fl	Raw, rippen	Mk011
*Carissa spinarum* L.	Apocynaceae	Agamsa	S	a, c, f	F	Raw, rippen	Mk004
*Colocasia esculenta* (L.) Schott	Araceae	Godare	H	j	R	Cooked	Mk081
*Cordia africana* Lam	Boraginaceae	Wadesa	T	a, b, c, d, e	F	Raw, rippen	Mk009
*Dovyalis abyssinica* (A. Rich.) Warb	Flacourtiaceae	Komsho	S	e	F	Raw, rippen	Mk018
*Dovyalis caffra* (Hook.f. and Harv.) Warb	Flacourtiaceae	Koshomi	S	e	F	Raw, rippen	Mk010
*Ekebergia capensis* Sparrm	Meliaceae	Sombo	T	a, b, d	F	Raw, rippen	Mk077
*Embelia schimperi* Vatke	Myrsinaceae	Hanku	S	a, d	F	Raw, rippen	Mk019
*Eriosema cordifolium* Hochest. ex A.Rich	Fabaceae	Kurkufo	H	c, f	R	Raw, chewing	Mk073
*Euclea racemosa* L.	Ebenaceae	Miessa	S	a, c	F, Tw	Raw, rippen, chewing	Mk014
*Ficus sur* Forssk	Moraceae	Harbu	T	b, c, d	F	Raw/dried, ripen	Mk008
*Ficus sycomorus* L.	Moraceae	Oda	T	b, c, d	F	Raw/dried, rippen	Mk021
*Ficus thonningii* Blume	Moraceae	Dambi	T	a, d, e	F	Raw/dried ripen	Mk075
*Ficus vasta* Forssk	Moraceae	Kiltu	T	b, c, d, e	F	Raw/dried rippen	Mk022
*Flacourtia indica* (Burm.f.) Merr	Flacourtiaceae	Huda	S	a	F	Raw, rippen	Mk021
*Gardenia ternifolia* Schumach. and Thonn	Rubiaceae	Gambelo	T	a, c	F	Raw, rippen	Mk024
*Grewia bicolor* Juss	Tiliaceae	Haroresa	S	a	F	Raw, rippen	Mk074
*Grewia ferruginea* Hochst.ex A.Rich	Tiliaceae	Dokonu	S	a	F	Raw, rippen	Mk020
*Grewia Villosa* Will	Tiliaceae	Dokonu	S	a	F	Raw, rippen	Mk012
*Impatiens rothii* Hook. f	Balsaminaceae	Ansosila	H	a, e	N	Raw, sucking	Mk028
*Justicia ladanoides* Lam	Acanthaceae	Dumuga	S	e, f, g	N	Raw, sucking	Mk082
*Lippia adoenesis* var. adoenesis Hochst. ec Walp	Verbenaceae	Koshonot	S	e	L, St	Spicing	Mk010
*Lippia adoenesis* var. *koseret* Hochst. ec Walp	Verbenaceae	Kusaye	S	a, c, e, f	L, St	Spicing	Mk078
*Mimusops kummel* Bruce ex A.DC	Sapotaceae	Koladi	T	a	F	Raw, rippen	Mk079
*Myrsine africana* L.	Myrsinaceae	Kachama	S	a	F	Raw, rippen	Mk066
*Olea europaea* L.	Oleaceae	Ejersa	T	a, b, c, e, f, g	F, L	Raw, rippen, Condiment	Mk042
*Phoenix reclinata* Jacq	Arecaceae	Meti	T	a, b, c, d	F	Raw, rippen	Mk026
*Physalis peruviana* L.	Solanaceae	Awuti	H	E	F	Raw, rippen	Mk027
*Rhamnus staddo* A.Rich	Rhamnaceae	Kadida	S	A	L	Condiment	Mk083
*Searsia glutinosa* (Hochst. ex A.Rich.) Moffett	Anacardiaceae	Tatesa	T	a, c	F	Raw, rippen	Mk003
*Searsia retinorrhoea* (Steud. ex Oliv.) Moffett	Anacardiaceae	Dabobesa	S	A	F	Raw, rippen	Mk076
*Rosa abyssinica* R.Br. ex Lindl	Rosaceae	Kakawe	S	a, c, h	F	Raw, rippen	Mk006
*Rubus apetalus* Poir	Rosaceae	Gora gure	S	a, c, d, h	F	Raw, rippen	Mk007
*Rubus steudneri* Schweinf	Rosaceae	Gora Arba	S	a, c, d, h	F	Raw, rippen	Mk006
*Rumex nervosus* Vahl	Polygonaneae	Dangago	S	a, c	L, St	Raw, chewing	Mk025
*Saba comorensis* (Bojer ex A.DC.) Pichon	Rubiaceae	Bururi	T	A	F	Raw, rippen	Mk080
*Sporobolus pyramidalis* P. Beauv	Poaceae	Muri	H	c, f	Se	Backed	Mk030
*Syzygium afromontanum* (F. White) Byng	Myrtaceae	Gosu	T	a, b, c, d	F	Raw, rippen	Mk012
*Syzygium guineense* (Wild.) DC. subsp*. guineense*	Myrtaceae	Badesa	T	a, b, c, d	F	Raw, rippen	Mk002
*Syzygium guineense* (Wild.) DC. subsp. macrocarpum (Engl.) F. White	Myrtaceae	Gumari	T	a, c, d	F	Raw, rippen	Mk029
*Vepris nobilis* (Delile) Mziray	Rutaceae	Hadesa	T	a	F	Raw, rippen	Mk069
*Thymus schimperi* Ronniger	Lamiaceae	Tosegn	H	c	L	Boiled	Mk049
*Urtica simensis* Hochst. ex A.Rich	Urticaceae	Sama	H	e, f, h	L,St	Cooked	Mk016
*Gymnanthemum amygdalinum* (Delile) Sch.Bip	Asteraceae	Ebicha	S	a, d, e, h	L	Condiment	Mk048
*Ximenia americana* L.	Oleaceae	Akuku	S	A	F	Raw, rippen	Mk072

H, habit (T, tree; shrub, S; H, herb; E, epiphyte); Hb, habitat (a, patchy forest; b, farm and arable land; c, pasture and grassland; d, riverine; e, home garden; f, field margin and roadsides f; g, woodlot; h, wasteland; i, on tree-trunk; j wetland j); Pu, Parts used (G, gum/exudate; Se, seed; F, fruit; L, leaf; St, stem; Fl, flower; R, root tuber; Y, young shoot; Tw, twing; N, nectar); Nn, voucher number

The highest number of these UWEPs was found in the family *Moraceae* (4 species, 8%), followed by *Fabaceae*, *Flacourtaceae*, *Myrtaceae*, *Rosaceae*, and *Tiliaceae* (each contributed 3 species, 6%). Those species in the family *Moraceae* were contributed by 4 genera (10.3%), in *Fabaceae* by 3 genera (7.7%), in *Flacourtaceae, Rosaceae,* and *Tiliaceae* each by 2 genera (5.1%), and in *Myrtaceae* by 1 genus (2.6%)*.* The remaining 7 families and 17 families each contributed two species (4% each) and one species (2%), respectively. Thus, 43.3% of the families were represented by more than one UWEP species. One or more of these families with the highest edible species contribution were consistently recorded in different ethnobotanical wild edible inventories [3, 7, 38, 40, 42, 46]. In particular, *Moraceaea, Fabaceae,* and *Tiliaceae* were mentioned for their highest number of wild edible resource contributions in different parts of the country [7]. The distribution could be attributed to their wider distribution throughout various agroecological zones all over the country. At the genus taxonomic level, the genera *Ficus* (4 species), followed by *Grewia* and *Syzygium* (3 species each), contributed the highest number of species. Either one or more of these highly wild food-contributing genera were also recorded elsewhere in Ethiopia [38, 40, 47].

The habitat distribution of the surveyed UWEPs covered a diverse ecological range from low to high land (1290–3058 m) above sea level. Explored habitat analysis showed that the species were recorded from a variety of habitat reservoirs. About 35 species (70.0%) were found in the forest, followed by pasture and grassland (24 species, 46.0%), riverine and home gardens (each 14 species, 28.0%), farm and arable lands (12 species, 24.0%), and the other possible habitat areas indicated in Table 2. A low diversity of plant species was recorded from the highlands, while a larger diversity of species was recorded from the midlands and lowlands (Fig. 3). This could be an indication that the local communities in these two agroecologies have retained more indigenous knowledge of their quoted plants. Similarly, the study conducted on prospects for sustainable use and development of wild food plants [3] and the ethnobotanical study of edible wild plants in Ensaro district, Amhara regional state [42] in Ethiopia indicated that the midland and lowland agroecology of the country were highly enriched by wild food plants, whereas the highland was known for low diversity, and areas with higher elevations are mostly known for their limited plant diversity [48].

### Underutilized wild edible plants habit and parts used

**Fig. 2:**
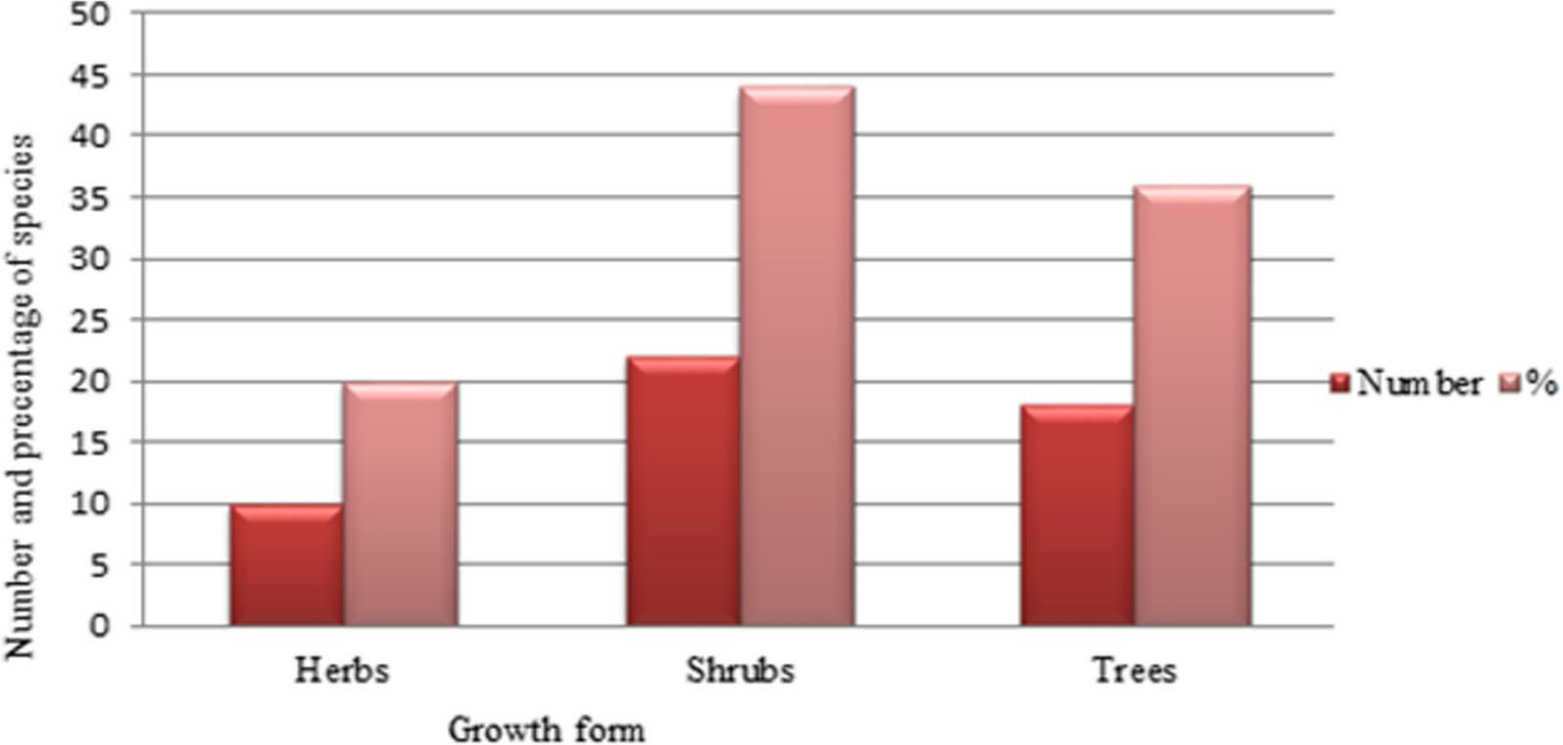
Habit of underutilized wild edible plants used in Midakegn District

Habit analysis of UWEPs used as edible food during different conditions in the study area revealed that shrubs constituted the largest category (22 species, 44%), followed by trees (18 species, 36%), whereas herbs consisted of the lowest life form (10 species, 20%) (Fig. 2). Our finding was agreed with the previous study reports [6, 37, 38, 40], which reveal that the predominant source of underutilized wild edibles was shrubs and trees. The review report analysis done by [7] at the country level also reveals that shrubs occupied the dominant position in contributing wild edible resources, followed by trees, herbs, and climbers. The study conducted in Nhema communal area, midlands province, Zimbabwe, by [49] also indicated that wild edible plant resources were mainly from trees and shrubs. In contrast to our study report, [50] reported the dominance of herbs followed by shrubs in an ethnobotanical study conducted in Burji District, Southern Ethiopia. On the other hand, the study conducted by [36], which indicated that trees, followed by herbs, contributed the most edible resources, also contrasts our findings.

**Fig. 3:**
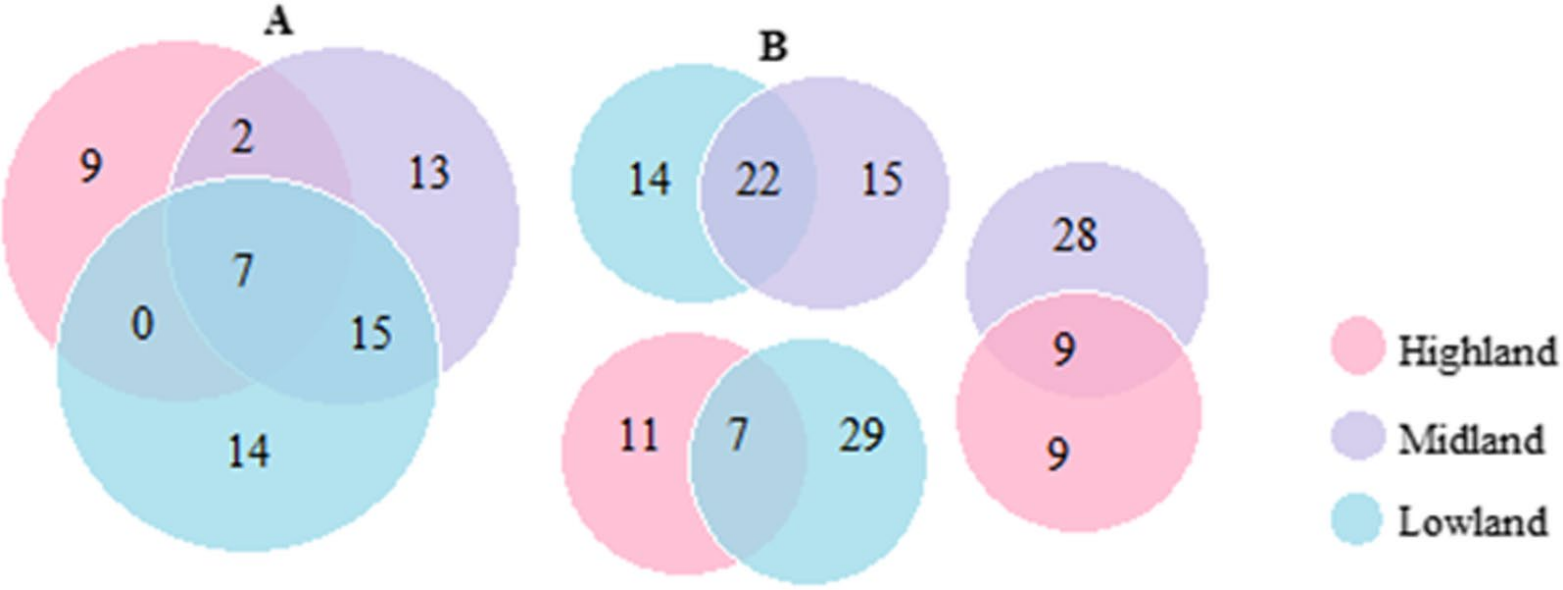
Venn diagrams show cross-comparisons of UWEP parts in use in the study area. **A** shows overall homogeneity and heterogeneity among the three agroecologies; **B** shows homogeneity between agroecologies

In this finding, a total of 53 UWEP parts were used as food in the study district under different circumstances. This includes fruits, leaves, leaves and stems, gums or exudates, seeds, young shoots, flowers, root tubers, nectars, and twings (Table 3 and Fig. 3). A smaller number of edibles (34%) were reported in the highland when compared to those reported in the midland and lowland of the study area (69.8% and 67.9 species, respectively). UWEPs commonly harvested for their fruits accounted for 33 species (62.3%), followed by plants harvested for their leaves and stems (5 species, 9.4%), and leaves alone (4 species, 7.5%), whereas those collected for other parts accounted for 20.8%. The study reports on UWEPs in the Chilga district, northwest Ethiopia, by [12] and in Tigray, northern Ethiopia, by [43] also indicated that fruits were the most commonly used parts of UWEPs. Similar to this finding, [7, 11, 20, 40, 46, 52] reported that fruits were the most widely used parts compared to the others. This is also similar to the report [4], in which fruits shared the majority of underutilized wild edible parts in the Asian Pacific region. The study report [53] in Indonesia also indicated that fruits, particularly those from the wild, are plentiful but less well known and underutilized. A cross-comparison of underutilized plant parts commonly reported in the three agroecologies has revealed a high degree of heterogeneity, and only a small proportion (7 species, 13.2%) of the food uses of certain recorded plant parts were commonly shared among the three agroecological areas (Fig. 3A). In between the three groups (Fig. 3B), maximum homogeneity was recorded between lowland and midland (22 species, 41.5%), followed by midland and highland (9 species, 17.0%), whereas minimum homogeneity was recorded between highland and lowland (7 species, 13.1%). This could be due to variation in altitudinal and other environmental factors that determine plant diversity in the environment. Different natural environments may lead to different plant utilizations [54]. As altitudinal variation increases, the common plant tax shared between the agroecologies of the study area decreases. This is why the minimum homogeneity of UWEP parts between lowland and highland and the relatively maximum homogeneity between lowland and midland and midland and highland were recorded in the study area. The remarkable heterogeneity in the use of wild edible plants among different groups could be referred to as the lack of common practice between different communities [48].

**Table 3:** Parts of underutilized wild edible plants used in the Midakegn District

Parts used	Lowland		Midland		Highland		District level	
	No	%	No	%	No	%	No	%
Fruit	25	47.2	21	39.6	7	13.2	33	62.3
Leaf	2	3.8	3	5.7	3	5.7	4	7.5
Leaf and stem	3	5.7	5	9.4	3	5.7	5	9.4
Gum or exudate	1	1.9	1	1.9	1	1.9	2	3.8
Seed	2	3.8	1	1.9	0	0.0	2	3.8
Young shoot	0	0	0	0	1	1.9	1	1.9
Flower	0	0	1	1.9	1	1.9	1	1.9
Root tuber	1	1.9	2	3.8	1	1.9	2	3.8
Nectar	1	1.9	2	3.8	1	1.9	2	3.8
Twing	1	1.9	1	1.9	0	0	1	1.9
Total	36	67.9	37	69.8	18	34.0	53

The local community uses 67.3% and 25% of the quoted edible parts to supplement staple food and as emergency food, respectively, whereas UWEP parts chewed during the drought and utilized to get relief trust each account for 3.8%. Edible plant parts from the wild are used as supplementary, seasonal, or survival food sources in various cultural groups in Ethiopia [7]. They support the rural livelihoods of the local community both during ample food production and during the need for emergency safety nets in conditions of food shortage, famine, and poverty [17, 37, 55] and hence play a role in combating food insecurity, especially for rural poor communities. They play a significant role in the subsistence and economy of resource-poor people throughout developing countries [18].

### Condition of preparation, form and mode of consumption

**Fig. 4:**
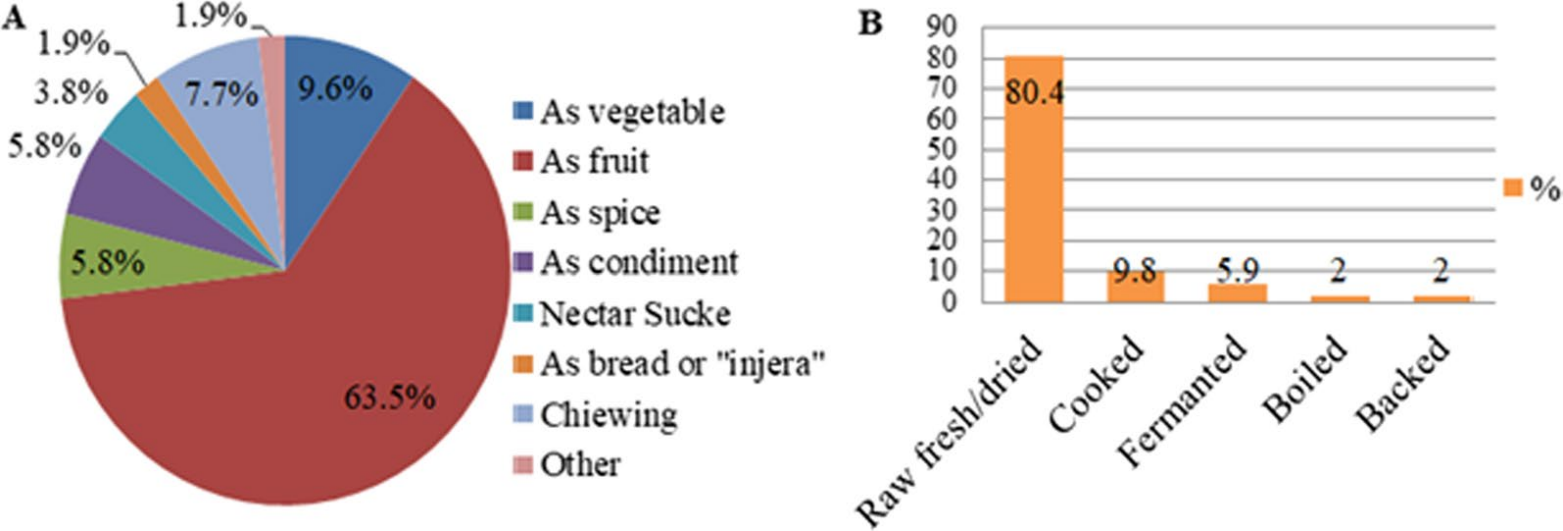
Consumption pattern of underutilized wild edible plants, **A** indicates the form of consumption, **B** indicates the mode of consumption

The local community quoted 53 plant parts from the total species reported in this study area. Out of these, 41 edible parts (77.4%) were directly consumed without further processing, whereas 12 plant parts (22.6%) needed further processing prior to use as food. Those edible parts were mainly consumed as fruits (62.3%), followed by those consumed as vegetables (9.6%). Others were consumed in the form of chewing (7.7%), spices (5.8%), condiments (5.8%), nectar sucking (3.8%), bread and/or “*Injera*” (1.9%), and other forms (1.9%). The main mode of consumption (80.4%) was direct utilization of raw fresh or raw dried edibles, followed by those consumed after cooking (9.8%) and fermentation (5.8%), respectively (Fig. 4). According to study findings from different regions of Ethiopia [6, 37, 40, 52], Zimbabwe [51], and Sudan [56], raw consumption was noted as the main way that people consumed wild edible foods.

According to the study area respondents, all the recorded fruits were directly consumed raw outdoors in the fields while working, keeping livestock, and traveling from place to place. The wide use of fruits is due to their ease of processing, more preferable taste, day-to-day requirements, and nutritional value [6, 38], the taste quality reduction during preservation, and the difficulty of preserving plenty of fruits for the indigenous people [49]. For instance, fruits of *S. afromontanum*, *S. guineense* subsp. *guineense*, *F. indicia*, *M. kummel*, *X. americana, R. steudneri, F. sur*, *D. abyssinica*, *R. apetalus*, *C. spinarium*, *R. abyssinica*, and *C. africana* relatively had more appreciation among the local communities and were consumed as supplementary food. In the review report [7], all of those species were listed among the common sources of edible fruits elsewhere in Ethiopia. The same is true for *C. africana* and *F. sycomorous* in Sudan [56]*.* There were also raw fruits, which received no more appreciation among the local community but served as supplementary food in the study area. Freshly ripened fruits of *G. bicolor*, *G. ferruginea*, *G. Villosa*, *E. racemosa*, *M. africana, P. peruviana*, *S. comorensis,* and *T. nobilis* were eaten raw outdoors by all community members except the fruits of *P. peruviana,* which were mainly considered children's food. However, harvesting of fresh edibles for consumption from some species, such as *F. vasta*, *F. sycomorous*, *E. kapensis*, *F. thinningii*, *G. terniffolia*, *R. glutinosa*, and *P. reclinata,* was most commonly to alleviate starvation during famine. In this case, both sexes and all age groups of both wealth classes, especially the elders, tried to access those edibles as alternative sources only during famine. This suggests that the majority of local communities continue to undervalue the potential use of the resources. But whenever edible resources were available, even when there was no scarcity of food, mostly youngsters and herdsmen search for collection and enjoyed consumption in the field. However, consuming excessive amounts of particular fruits, such as *F. sura*, *F. vasta*, *F. sycomorus*, and *F. thonningii*, whether raw or dried, has been linked to gastrointestinal discomfort. Similar to this, [46] stated that in the region of Konso ethnic communities in South Ethiopia, stomach pain and diarrhea are common health problems following the consumption of numerous wild edible plants. Fruits were not the only edibles eaten raw; other parts were also utilized raw and fresh without needing further processing. During times of drought, the local population chewed raw gums of *V. abyssinica* and *F. albida* as well as fresh, just emerged lateral and terminal shoots of *R. nervosus*, mostly to reduce their need for water. Underground root tubers of *E. cardifolium* were chewed raw by children, particularly when keeping cattle. Children in the study area also used the flowers of *I. rothii* and *J. schimperiana* to suck the nectar that provided them energy. A fresh flower cavity of *C. eminii* was filled with either fruits of *R. steudneri, R. apetalus*, or *R. abyssinica*, which were consumed together by children in the study area. In addition to the fruits consumed as supplementary food, twigs of *E. racemosa* were chewed by all community members to get refreshment.

On the other hand, only 22.6% of edible parts that needed cooking, backing, condimenting, and spicing were brought home before dish preparation and were consumed by all family members in the home. This result agreed with the finding of [52], in which a few wild edible plant parts were brought home for cooking before consumption, and dishes prepared from them were consumed by entire groups [57]. But cooking responsibilities were accomplished by women and female youngsters in the study area. For instance, the collection and preparation of the leaves of *A. hybridus*, the leaf and stem of *U. simensis,* and the young shoot of *O. alpina* as vegetables mainly during food scarcity were done by women and young girls. Even though it was a very rare species in the study area, the root tubers of *C. esculenta* were also cooked by women and consumed as vegetables in the study area. Similarly, powdered grain seeds of *S. pyramidalis* were backed into bread, or "injera," just like *Eragrostis tef* (Zucc.) Trotter flour and seeds of *F. albida* were cooked into boiled grain, or "*Mulluu*," b*y* women and young girls and consumed by all family members during the famine. Women also mainly participated in the leaf collection of *O. europaea L.* subsp*. cuspidata, R. staddo,* and *G. amygdalinum* for use as additives in the fermentation process of different local alcohols, such as "areke,*"* "tela,*"* and "teji*".* Also, commonly, harvesting the leaves of *L. adoenesis* and *T. schimperi* and using them in the spicing or flavoring process was done by women. The review report done on the contribution of indigenous food preparation and preservation techniques for the attainment of food security in Ethiopia [57] indicates that leafy wild edible material collection and processing for consumption are mostly considered the responsibility of women and young girls in Ethiopia. The leaves and stems of *L. adoenesis* var. *adoenesis* and *L. adoenesis* var. *koseret* were used for various food additive spicing purposes (e.g., pepper powder, butter, etc.), and *T. schimperi* was used as tea flavoring. Milk pots and other kitchen equipment were also cleaned and fragranced with the leaves of the two *L. adoenesis* varieties in the study area. The Gurage and Oromo peoples use the fragrant leaves as one of the spices for making spiced butter [35]. Food prepared with spiced butter and/or spiced pepper powder had a delicious flavor that attracted people to eat it. In addition to all these recorded underutilized edibles from the wild that were used to maintain the sustainability of food security and food sovereignty in the study area, they have more contributions than the locals are aware of. Especially for the local poor, their utilization not only assures food security but also supplies vital nutrients that prevent malnutrition. As [58] reported, consumption of wild edible fruit provides more nutritional value, such as vitamins, fibers, and secondary metabolites, to the human diet than cultivated crops, and they have a good content of minerals (cupper, magnesium, and phosphorous), carotenoids, and protein. The nutritional composition analysis done on underutilized edible fruits of *Balanites aegyptiaca* (L.), *Grewia flavescens* Juss., and *Ziziphus spina-christi* Willd. also indicated that their fruits are enriched with major food substances such as carbohydrate, crude protein, crude fat, and minerals [17]. On the other hand, proximate amino acids, minerals, and ant-nutritional factors analysis of popularly consumed fifteen wild edible plants in Hamar and Konso of southern Ethiopia also revealed that wild leafy vegetables contribute good amounts of these essential nutrients to the human diet [59]. The local community not only benefited from the utilization of these underutilized edible species but also used them for multiple purposes. As an example, active substances from edible parts of *E. schimperi*, *G. amygdalina*, *L. adoensis*, *O. europaea*, and *R. nervous* include those that are utilized as traditional remedies. *E. schimperi* fruit was traditionally used to treat tapeworms after the powdered or crushed fruits were mixed with water and taken orally. *G. amygdalina* leaves were chewed, and the juice was then swallowed internally to treat bronchial infections. *L. adoensis's* squeezed and filtered leaf extract was administered orally to treat fibril illness, and the afflicted portion of the eyelid was also directly rubbed with the leaves to cure eye infection. A similar ethnopharmacological importance of these species was also reported by [60]. Leaf extract of *G. amygdalina added* to local drinks was also used for treating stomach problems in lowland areas of Ethiopia [61]. The water-mixed, pounded leaf buds of *O. europaea* were used to protect intestinal parasites. The leaves of *R. nervous* were smashed and directly rubbed over the skin hemorrhage to treat the infected body in the study area.

### Marketability of underutilized wild edible parts in the study area

Other than food service, some of the UWEPs in the study area were used as an alternative to supplement household income. The conducted market survey and information assessed from informants revealed that 17% of total UWEP parts recorded in the district were sold in the local market (Table 4). Mostly youngsters and sometimes women of the poorest wealth class were observed and reported as the most sellers of edible parts of these species. Similarly, young children in the study report [11, 20] were mentioned as the main selling group of wild edible plant parts, especially fruits. Women and children of the Nhema communal area of Zimbabwe were also reported as common sellers of wild edible parts in the local market [51]. An example and the most frequently cited species for their fruits are *S. afromontanum,* followed by *S. guinense* subsp*. guinense, F. indicia, M. kummel,* and *X. Americana* (Table 4)*.* Currently, some fruits of these species are sold for 2–5 Ethiopian birr ($\approx$ 0.037–0.092 USD) per cup or plastic glass in the local market of the study area and were sold for fewer prices for one to three decades before. In agreement with this finding, the fruits of *S. guinense* and *X. americana* in the study conducted by [37], *S. guinense* and *M. kummel* by [38], and *M. kummel*, *S. guineense*, and *F. indica* [44] were also used for income generation in other parts of Ethiopia. Leafy shoots of *L. adoenesis* var. *adoenesis, Lippia adoenesis* var. *koseret,* and leaves of *T. schimperi* are currently sold in the local market for spicing purposes.

**Table 4:** Marketability of the underutilized edible parts in the study area

Species	MP	MU	PR	SG	WC	NR
*F. indica*	Fruit	Cup/glass	2–5 ETB	Y	Poor	31
*L. adoenesis var.adoenesis*	Leaf and stem	Can/jug	10–20 ETB	Y and W	Both	23
*L. adoenesis var. koseret*	Seedling, Leaf and stem	Number, handful	5 ETB	Y and W	Poor	13
*M. kummel*	Fruit	Cup/grass	2–5 ETB	Y	Poor	29
*R. steudneri*	Fruit	Fruit inflorescence	2–5 ETB	Y	Poor	24
*S. guinense* subsp. *guinense*	Fruit	Cup/glass	2–5 ETB	Y and W	Poor	39
*S. afromontanum*	Fruit	Cup/glass	3–5 ETB	Y and W	Poor	55
*T. schimperi*	Leaf	Can/jug	5–10 ETB	W	Both	17
*X. americana*	Fruit	Cup/glass	5 ETB	Y and W	Poor	25

MP, marketable part; MU, measuring unit; PR, price in Ethiopian birr (ETB), 1ETB $\approx$ 0.0184 USD; SG, seller group, WC, wealth class; NR, number of respondents; Y, young; W, women

### Preference ranking of underutilized wild edible plants and threatening factors

**Fig. 5:**
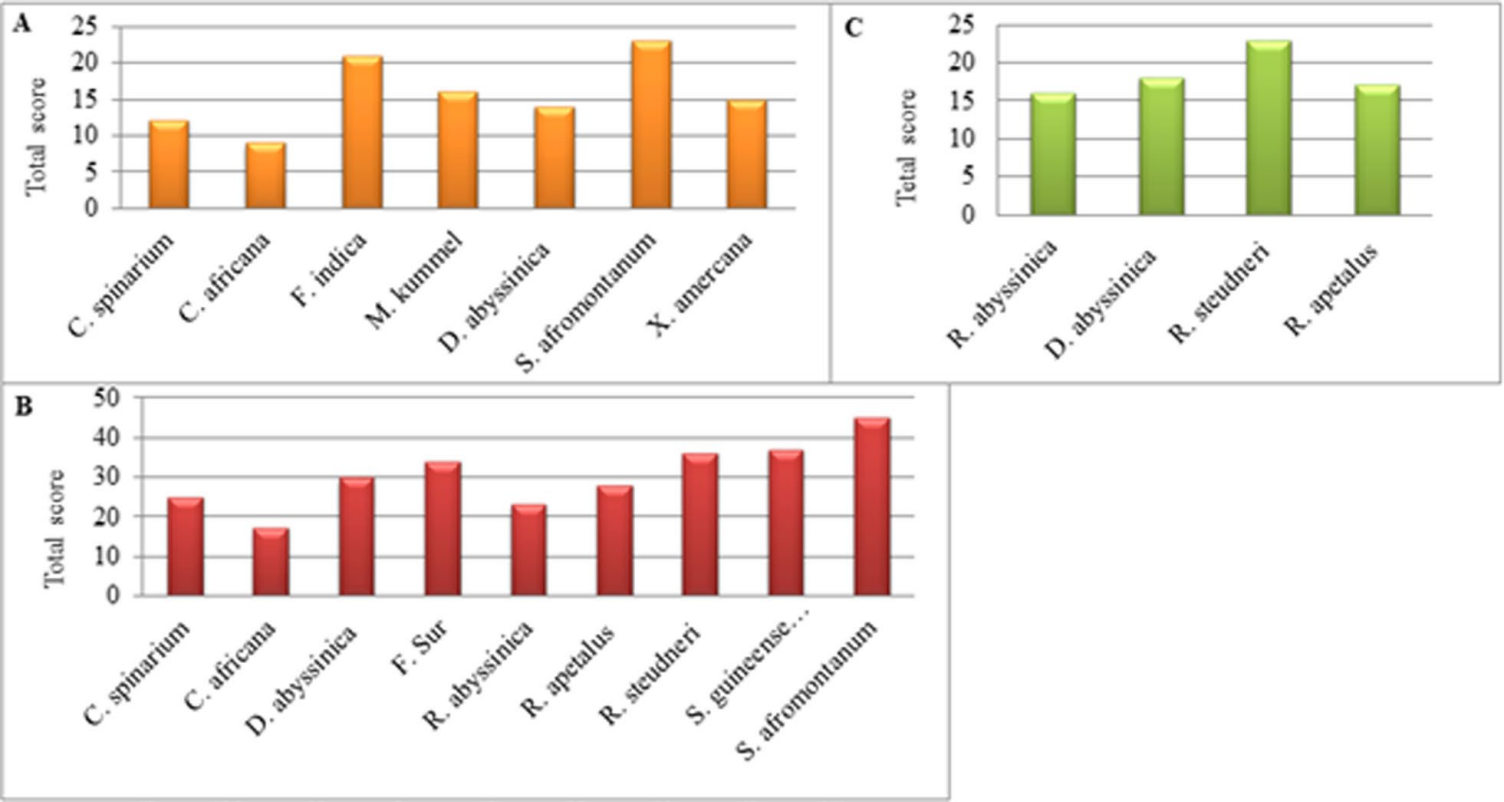
Preference ranking of underutilized wild edible parts based on taste quality and frequency of citation at **A** lowland, **B** midland, and **C** highland agroecological areas

The determinant factors of the preference status of one edible plant over another were commonly based on taste, availability, accessibility, cultural, psychological, or inherited ancestral practices [17, 51]. In this study, based on taste quality perceived by KIs and frequency of citation, preference ranking for 7, 9 and 4 underutilized wild edible foods at low, mid, and highland agroecologies of the study areas was carried out to find out their relative importance to the local community (Fig. 5). The ranks were given by each selected KI from each agroecology at each study site. The total score rank of 10 KIs at midland indicates the most preferred species, in descending order, were *S. afromontanum*, *S. guineense* subsp. *guineense*, *R. steudneri*, *F. sur, D. abyssinica, R. apetalus, C. spinarium, R. abyssinica,* and *C. africana*. Compared to the others, *S. afromontanum* got the highest score (total score = 45) due to its best palatability and was used as a supplementary food in the midland study site. *S. guineense* subsp. *guineense,* which scored second (total score = 37), was used for the same purpose with a better and more pleasant taste; *R. steudneri,* which scored third (total score = 36), was also used as supplementary food with a better and more suitable taste, and *F. sur*, which was used as supplementary food, scored fourth (total score = 34) and had a good taste. *D. abyssinica,* which was scored fifth, and *R. apetalus,* which was scored sixth, got a total score of 29 and 28, respectively, and they serve as supplementary foods with somewhat good taste quality. The supplementary edibles C*. spinarium* was score seventh (total score = 25), and *R. abyssinica* was scored eighth (total score = 23) with fair taste quality*. C. Africana* was scored ninth (total score = 17), which indicates it has the least taste quality. Accordingly, the total score and the rank were done for those edibles from lowland and highland study sites accordingly, and the preference score rank of seven UWEPs done by five KIs at lowland agroecology in descending order also indicates *S. afromontamum, F. indica, D. abyssinica, R. apetalus, C. spinarium, M. kummel,* and *C. africana* are among the top 7***.*** Another rank score done for four species with five KIs at highland agroecology showed more preference for *R. steudneri* over the others, followed by *R. apetalus*, *R. abyssinica,* and *D. abyssinica.* The sequence of ranks at each agroecology was indicated by total score, and edible with the highest total score has better palatability than the next and is hence more preferable to the one with the next highest total score.

The causes of threats to UWEPs can be generally grouped into anthropogenic and naturally induced factors. Human-induced factors recorded by the informants as local threats to UWEPs were agricultural land expansion, fuel wood harvesting, selective harvesting for various purposes, overgrazing, urbanization, and fire, and the natural-induced factors recorded were drought and land degradation. The preference ranking exercise helped to prioritize the most threatening factors affecting such resources in the study area, indicating agricultural land expansion, overgrazing, and fuel wood collection (harvest for charcoal and firewood) ranking 1st, 2nd, and 3rd, respectively, whereas the destructive effects due to selective harvesting for different purposes (construction materials, farm and household tools, traditional medicine, fumigate material), fire hazards, other natural disasters (such as drought, land degradation, etc.), and urbanization held 4th–7th ranks in the study area, respectively (Table 5). Even though the above threatening factors were ranked at the district level, the destructive effect of each factor varies among informants from the three agroecological zones of the district. For instance, KI1–KI3 in Table 5 were from the lowlands, and thus, the individual values given to agricultural expansion, fire, and overgrazing indicated that they were the principal threats to UWEPs in the lowland agroecological zones of the study area. This is mainly due to increasing demand for farmland and a large mass of livestock moving from highland and midland to the area for grazing purposes. Searching for newly vegetating grass and to protect against animal attack (e.g., snakes), people released fire either deliberately or unintentionally to burn the grassland in the study area. Similar findings and reasons for wild edible plant habitat destruction and conversion were reported in other parts of Ethiopia [50, 62]. The causes of threats ranked 1st, 2nd and 4th in this study were also reported as the principal factors for wild food plants in the Eastern Nuba Mountains, Sudan [56]. The recent rate of wild edible plant habitat overexploitation for various human needs was indicated as a big threat to the long-term existence of those resources in Ethiopia [50]. As observed in the study area, mainly in the highland and midland, those main destructing factors had a significant impact on particular wild edible plants, especially those used for multiple uses. For example, *O. europaea* subsp. *cuspidata*, *E. kapensis*, *C. africana*, *V. abyssinica*, *S. guineense* subsp. *guineense*, and *F. sur* were observed as a single tree either in cultivated land, around home gardens, or riverine, but were rarely observed in the small patchy forests.. The situation is definitely linked up with the need for excessive yield production from a few staple crops to balance the demand of an ever-increasing population, overharvesting for multiple uses, and a lack of full understanding of the nutritional, economic, sociocultural, and ecological importance of indigenous wild edible plants and their associated indigenous knowledge to current and future generations. Thus, more strategies have been needed for such species that need conservation priority than the less potential plant biodiversity in the study area.

**Table 5:** Priority ranking on recorded threatening factors to underutilized wild edible plants

Threatening factors	10 Key informants											
	L1	L2	L3	M1	M2	M3	M4	H1	H2	H3	Total	Rank
Agricultural expansion	5	5	5	5	6	6	6	5	5	6	54	1st
Fuel wood harvest	3	5	4	5	4	4	3	4	3	3	38	3rd
Selective harvesting	4	3	3	3	4	4	3	3	4	3	34	4th
Over-grazing/stocking	5	4	5	4	5	3	4	3	4	5	42	2nd
Urbanization	1	1	2	1	1	1	1	1	1	1	11	7th
Fire	5	5	5	2	2	3	3	3	2	2	32	5th
Other natural disasters	2	1	2	2	2	1	2	2	1	2	17	6th

L1**–**L3, lowland; M1**–**M4, midland; H1**–**H3 highland key informants

### Use diversity of the UWEPs in the study area

A total of 50 UWEPs documented in the study area had multiple uses other than food value. Of these, 2 species (4%) were mainly used for consumption only, while 48 species (96%) had multiple uses in addition to their edibility. The multiple uses include: medicinal value accounted for by 23 species (46%), construction by 18 species (36%), fuel wood by 32 species (64%), bee forage by 14 species (28%), fodder by 32 species (64%), farm and household tools by 22 species (44%), live fence by 12 species (24%), live shade by 12 species (24%), soil and water conservation by 11 species (22%), and other use categories (spicing, flavoring, condemning, fumigating, and toothbrush) were by 10 (20%) species. However, other than the edibility function, direct matrix ranking was done for ten very common and frequently reported UWEPs under nine use categories (etic categories) (Table 6), and the result showed that *O. europaea* subsp. *cuspidata, C. africana, V. abyssinica, S. guineense* subsp. *guineense, and S. afromontanum* were found to be most important in their multiple utility values, respectively. This indicates that these UWEPs were more exploited for their multipurpose function than the others in the study area. Overharvesting of UWEPs for various exploiting uses such as fuel wood, construction, farm, and household tools were found to be the responsible threatening factors aggravating the depletion of the species. Similarly, [11] said that the reason for the highest exploitation of wild edible plants was because of their multiple uses other than their food values. Thus, such species need urgent complementary conservation action and sustainable use to save and manage the fast-eroding multipurpose UWEPs in the study area.

**Table 6:** Average score of direct matrix ranking of ten UWEPs with different use values other than edibility in the study area

Use category	Species									
	*V. abyssinica*	*C. spinarium*	*C. africana*	*E. racemosa*	*F. sur*	*F. sycomorus*	*F. vasta*	*O. europaea*	*S. afromontanum*	*S. guineense*
Medicinal	3	5	3	3	3	2	3	4	2	2
Fuel wood	5	3	4	4	2	3	3	5	4	4
Construction	2	1	5	2	3	2	3	5	4	4
Farm and household tools	3	0	4	3	1	1	1	5	2	3
Fodder	3	2	3	2	4	3	4	3	3	3
Shade	4	0	3	1	5	4	5	3	3	3
Live fence	2	1	3	2	0	0	0	4	1	1
Bee forage	3	2	5	3	0	0	0	3	5	5
Soil and water conservation	5	2	4	1	4	4	4	3	3	3
Total score	30	16	34	21	22	19	23	35	27	28
Rank	3	10	2	8	7	9	6	1	5	4

### Knowledge distribution of underutilized wild edible plants between different informant groups

The mean comparison of knowledge distribution among different informant categories is indicated in Table 7. The average comparison between different informant groups indicates that significantly higher (*P* < 0.05) numbers of UWEPs were claimed by KIs than GIs, by elder respondents (age > 40 years) than those reported by young to middle-aged adults (18–40 years), by males than females, and by illiterate groups than literate groups.

**Table 7:** Mean comparison of the numbers of underutilized wild edible plants reported by different informant groups in Midakegn District

Parameters	Informant categories	Number	Mean ± SD
Age	18–40 years old	159	5.99 ± 2.02
	> 40 years old	199	9.06 ± 2.75
Informants	Key informants	25	11.72 ± 3.03
	General informants	333	7.39 ± 2.64
Sex	Female	67	6.70 ± 2.76
	Male	291	7.92 ± 2.87
Education level	Illiterate (0–4 grade)	268	8.06 ± 2.93
	Literate (> 4 grade)	90	6.59 ± 2.46
Economic status	Poor	116	10.03 ± 2.73
	Medium	129	6.76 ± 2.25
	Rich	113	6.36 ± 2.89

A significant difference (*P* < 0.05) between the means of the different categories

The results showed that knowledge distribution was almost unequally shared among different informant groups. As the age of informants (both male and female) increases, the level of their indigenous knowledge of UWEPs significantly increases. This could be linked to the opportunities they experienced with new wild edible resources under different climatic conditions. The study conducted by [20, 37] also indicated that the elder members of the community had higher ethnobotanical knowledge than the youngsters. But contrary to their knowledge level, youngsters have been consuming more than senior age groups in the study area. This is because children have an intimate association with wild edible fruits throughout the year [63], and they have more initiation to harvest the resource than adults [64].

On the other hand, the male informant group quoted more UWEPs than the female informant group. This could be linked with the norm and cultural influence of the study area in that, in day-to-day life activities, females were mostly restricted to working at home and in the home garden rather than in the field. Gender role stereotyping was reported as an ethnobotanical knowledge level determinant factor between males and females [20]. Male responsibilities such as harvesting wild edible plants for construction, agricultural tools, technologies, and household uses could let males have better knowledge than females [11].

Illiterate people in the study area reported more UWEPs than literate, which could probably due to the higher influence of modernization and distance from interaction to natural vegetation on the later informant groups. Another UWEP's citation analysis between economic status groups indicates that there was a significant difference between the poor and other wealth classes. This means UWEP's citation of the poor category was significantly higher (*P* < 0.05) than the medium and rich wealth categories in the study area. However, the citation of UWEPs in the medium and rich wealth categories didn’t show a significant difference (*P* > 0.05). A similar study reported by [12] indicates that local people with low economic status cited more wild edible plants than the others. More dependence of low livelihood communities on wild plants might be to ensure food security under different circumstances rather than they have been considered that have better nutritional value. Reliance on wild edible plants was greater in households with food insecurity that lacked off-farm income and had lower levels of assets [55]. Those local community members with better income and sufficient grain for food in any circumstance consume less wild edible plant foods and are taken as famine or low-class food [65]. Lack of awareness forced them to underestimate these plant resources, both nutritionally and socioeconomically, as they have less value than other cereals and pulses. Even when a serious food shortage affects all strata of a population, poor families regularly collect and consume wild food more than the richest families in different parts of Ethiopia [9].

The assessed information reflected that indigenous knowledge related to UWEPs has been passed down from generation to generation through oral transmission in families and neighborhoods. However, it has shown a declining trend and become out-dated due to the lack of appreciation by younger generations because of a shift in attitude and on-going socioeconomic changes in the study area. Such phenomena could result in both the eradication of wild food culture and its associated indigenous knowledge [53], and their inheritance is faced with great risks [49].

As information assessed from 25KIs indicates, consumption of edible parts from UWEPs is currently much reduced when compared to the past. Nineteen KIs (76%) of them reported that the reason behind the consumption decline of UWEPs in the study area is an increase in staple food crop production and community reliance on a few plant products to sustain their livelihoods. According to [17], farmers' intentions toward better cultivars, modernization, acculturation, and a lack of knowledge about the advantages of both native wild edible plants and their associated indigenous knowledge may lead to the reduction or loss of the resource and indigenous knowledge in the near future. Under normal circumstances, the contribution of wild edible plants to the overall food supply for the society of Ethiopia is relatively small and is simply utilized as supplementary or occasional snacks during certain periods [3], and except in a few southern part, the majority of the country has often perceived its consumption as a sign of poverty [59]. The globalization of agricultural marketing has become the most constraint to the promotion of UWEPs on a global scale [68]. Reduction in utilization may gradually cause the removal of indigenous knowledge associated with the species and thus pose a danger to low-income people who are relatively more reliant on these cheap foods.

On the other hand, 13 KIs (52%) mentioned that the UWEP consumption decline was due to the difficulty of getting edible materials at near distances under current climate conditions. Lack of awareness of their nutritional function was also mentioned by 3 KIs (12%) as another impact factor that has caused a reduction in the utilization of wild edible parts in the local community. An increased distance traveled to harvest edible materials by the collector and difficulties accessing them, as well as an increase in economic status, significantly correlate to lower levels of WEP use [55, 67]. On the other hand, wild plants have been affected by climate variability, and many of the plants have disappeared [67]. The continuous use of different plant species in a sustainable manner has been questioned as a result of land degradation and the worsening of climate changes from time to time [68].

### Management and conservation practice to underutilized wild edible plants

Information from both discussants revealed that there was no measurable participatory action implemented on the part of governmental and non-governmental agents to engage the local community to scientifically improve management practices for the conservation and utilization of UWEPs. Even at the country level, no conservation action or programs that support efficient utilization of wild edible plants have been undertaken [3]. However, 19 (38%) UWEPs were conserved by local communities through three traditional practices. One of the strategies informed and observed in the study area was culturally protecting plants in their natural environments because of their multiple uses. For instance, *V. abyssinica*, *F. albida, C. africana, F. sur, F. vasta, F. cycomorus, and E. capensis* were left as a single tree in the farmlands, farm boundaries, and watershed areas due to their capability of soil conservation, fertility improvement, and frequent use as a shade. Cutting trees such as *F. cycomorus* were strictly prohibited by community norms because it was considered a seating area for community elders to solve different conflicts in the community. The second management strategy was leaving UWEPs in farmland or around the home garden for their pollarding and re-pollarding nature, which allows the plants to have more branches for different construction services (e.g., *O. europaea*). Planting and keeping UWEPs around the home garden for their diverse uses were found to be the third management strategy practiced for conservation. A few species were planted in and around the home garden for their condiment function in local alcohol drink preparation and spicing function (e.g., *G. amygdaninum* and *L. adoenesis* var*.*). Others, such as *D. caffra* and *J. ladanoides,* were semi-cultivated for their life-fencing services, and *O. alpina* for its construction material provision. Similar management strategies were also reported in other parts of Ethiopia for the conservation and enhancement of multipurpose wild edible plants and for the preservation of the indigenous knowledge associated with them [37, 47]. However, very limited management activities were practiced when compared to those enacted for other staple food plants.

## Conclusions and recommendations

The present study revealed that Midakegn District is endowed with diverse UWEPs and associated indigenous knowledge. Fifty UWEPs belonging to 38 genera and 30 families were collected and documented in the study area. Nevertheless, a variety of their habitat types were recorded, and the majority of them were collected and distributed in the patchy forests. The analysis of the information from the discussant and interviewers showed that underutilized wild edible materials collected from shrubs and trees made the largest contribution to the local community and were consumed to supplement the staple food, as emergency food to get relief, and chewed during drought. Relatively, the significant contribution of UWEPs to the regular diet was greater for the poorest wealth class than the medium and higher classes in the study area. Other than food value, most UWEPs have multiple uses, such as medicinal, fuel wood, construction, farm and household tools, fodder, bee forage, live shade, life-fence, and soil and water conservation. Thereby, income is generated from the sale of their edible and other parts. Priority ranking based on recorded threatening factors for UWEPs indicates that many of the species are under growing pressure, mainly from various human-induced factors. They suffer from destruction for different uses, including agricultural land expansion, fuel wood harvesting, selective harvesting, overgrazing, urbanization, and fire. Natural disasters and a lack of intervention from the government and other bodies for scientifically managed action are also threating the sustainability of UWEPs. The local community only practices traditional management strategies for conservation action in the study district. Even though the current study emphasized on the documentation of UWEP species diversity along with their potential function in socio-economic activities, indigenous knowledge associated with them, and threats to them, the negligence to use UWEPs, which is fully linked with a lack of awareness of their nutritional and economic value, should be minimized. Thus, community awareness through training and further studies on the nutritional content analysis and economic valuation of promising UWEPs are needed. This could help the stockholders (consumers) realize the benefits of UWEPs, which in turn could encourage policymakers and investigators to optimize and promote the benefits. These actions might encourage domestication and further conservation of promising UWEPs through integration into existing land use types and, thus, ultimately have positive impacts on the future livelihoods of rural communities.

## Data Availability

The data are presented in tables and figures in this article. However, please contact the corresponding author for further inquiries requested.
